# Shigellosis in Nepal: 13 years review of nationwide surveillance

**DOI:** 10.1186/s41043-016-0073-x

**Published:** 2016-11-04

**Authors:** Geeta Shakya, Jyoti Acharya, Shailaja Adhikari, Nisha Rijal

**Affiliations:** National Public Health Laboratory, Kathmandu, Nepal

**Keywords:** *Shigella*, Antimicrobial resistance, Nepal

## Abstract

**Background:**

*Shigella* is a major cause of gastroenteritis especially in children. In developing countries, the incidence is frequent and results are often life threatening. Changing epidemiology and emerging antibiotic resistance warrants continuous monitoring of susceptibility. The present study highlights the changing epidemiology and drug resistance patterns of *Shigella* isolated at different hospitals of Nepal over a period of 13 years (Jan. 2003–Dec. 2015).

**Methods:**

This study was carried out in 12 participating laboratories. Stool specimens received at respective laboratories were processed for isolation and identification of *Shigella* species and confirmed by serotyping at National Public Health Laboratory. Antimicrobial resistance patterns were determined by Kirby Baeur disc diffusion test.

**Results:**

A total of 332 isolates were identified as *Shigella species* of which *Shigella flexneri* (50 %) was the predominant serotype. *Shigella dysenteriae*, *Shigella sonnei*, *Shigella boydii*, and untypable *Shigella* spp. respectively, accounted for 28.6, 27.54, 10.2, 4.5, and 6.6 % of the total number. Change in prevalent serotype is noted over the years. *S. dysenteriae* was the prevalent species in Nepal in 2003 and 2004, but since 2005, *S. flexneri* remained prevalent. Majority of the isolates were recovered from children aged 1–10 years and was statistically significant (*p* = 0.023) compared to the other age groups. High resistance among all *Shigella* species to the first-line drugs like ampicillin (88 %), cotrimoxazole (76 %), ciprofloxacin (39 %,) and nalidixic acid (80 %) was observed; 46.1 % of total isolates were multidrug resistant (MDR), and the most common MDR profile was ampicillin, nalidixic acid, and co-trimoxazole. Prevalence of MDR increased significantly in 2010 as compared to 2003. Only few *Shigella* isolates were resistant to ceftriaxone.

**Conclusions:**

The study revealed *S. flexneri* as the predominant serogroup in Nepal. Children below 10 years were more prone to the disease. Nalidixic acid, ampicillin, co-trimoxazole, and ciprofloxacin should not be used empirically as the first-line drugs in treatment of shigellosis. Since the distribution of different species of *Shigella* and their antibiotic susceptibility profile may vary from one geographical location to another and may also change with time, continuous local monitoring of resistance patterns is necessary for appropriate antimicrobial therapy.

## Background

*Shigella* is one of the most important etiological agents of diarrhea, in particular, dysentery commonly known as bacillary dysentery. It is a more severe form than gastroenteritis and is responsible for childhood morbidity and mortality, especially in developing countries [1–4]. Ingestion of even 100 microorganisms leads after 4–7 days to an acute diarrhea. Because of delay in humoral responses, complication and mortality rate due to shigellosis in children is higher than in other age groups [5]. A literature review concluded that of the estimated 165 million cases of *Shigella* diarrhea that occur annually in the world, 99 % occur in the developing world and the remaining 1 % occurs in industrialized countries. In developing countries, 69 % of these episodes occur in children under 5 years of age. Moreover, of the 1.1 million deaths attributed to *Shigella* infections in developing countries, 60 % occur in the under 5 age group in children [6].

The genus *Shigella* comprise of four different species which are referred to by a letter designation based on their serological antigen: serotype A-*Shigella dysenteriae*, Serotype B-*Shigella flexneri*, Serotype C-*Shigella boydii* and Serotype D-*Shigella sonnei*. The first three are common in developing countries while *S. sonnei* is common in developed countries and Iran [7].

Shifts in the prevalent serogroups have been observed in many parts of the world and so is true for Nepal. Studies conducted during1999–2002 shows high incidence of *S. dysentriae* infection in Nepal [8]. Studies conducted after 2002 showed prevalence of *S. flexneri* strains among *Shigella* isolates from all parts of Nepal [9–12].

According to WHO report, antimicrobial resistance pattern for *Shigella* varies in different parts of the world and with the time. Over the past decades, *Shigella* species have become progressively resistant to most widely used antimicrobials [13, 14]. Since the late 1990s, the fluoroquinolones, including ciprofloxacin, norfloxacin, and ofloxacin, have been the drug of choice for multidrug-resistant *Shigella* infections. However, strains resistant to the first-line drugs commonly used in the treatment of shigellosis like ampicillin, cotrimoxazole, and nalidixic and even ciprofloxacin and norfloxacin has also been reported from various studies in Nepal and worldwide [9–12, 15]. Ceftriaxone has been used as a reserved antibiotic for treatment of multidrug-resistant (MDR) *Shigella* infection [16]. However, some studies have also reported emerging ceftriaxone resistance in *Shigella*. This increasing level of antimicrobial resistance in *Shigella* have limited the therapeutic options and complicated the treatment of shigellosis.

The purpose of the present study is to determine the trend of change in *Shigella* species and their antimicrobial resistance patterns over a decade for the better management of shigellosis.

## Methods

### Surveillance sites

**Fig. 1:**
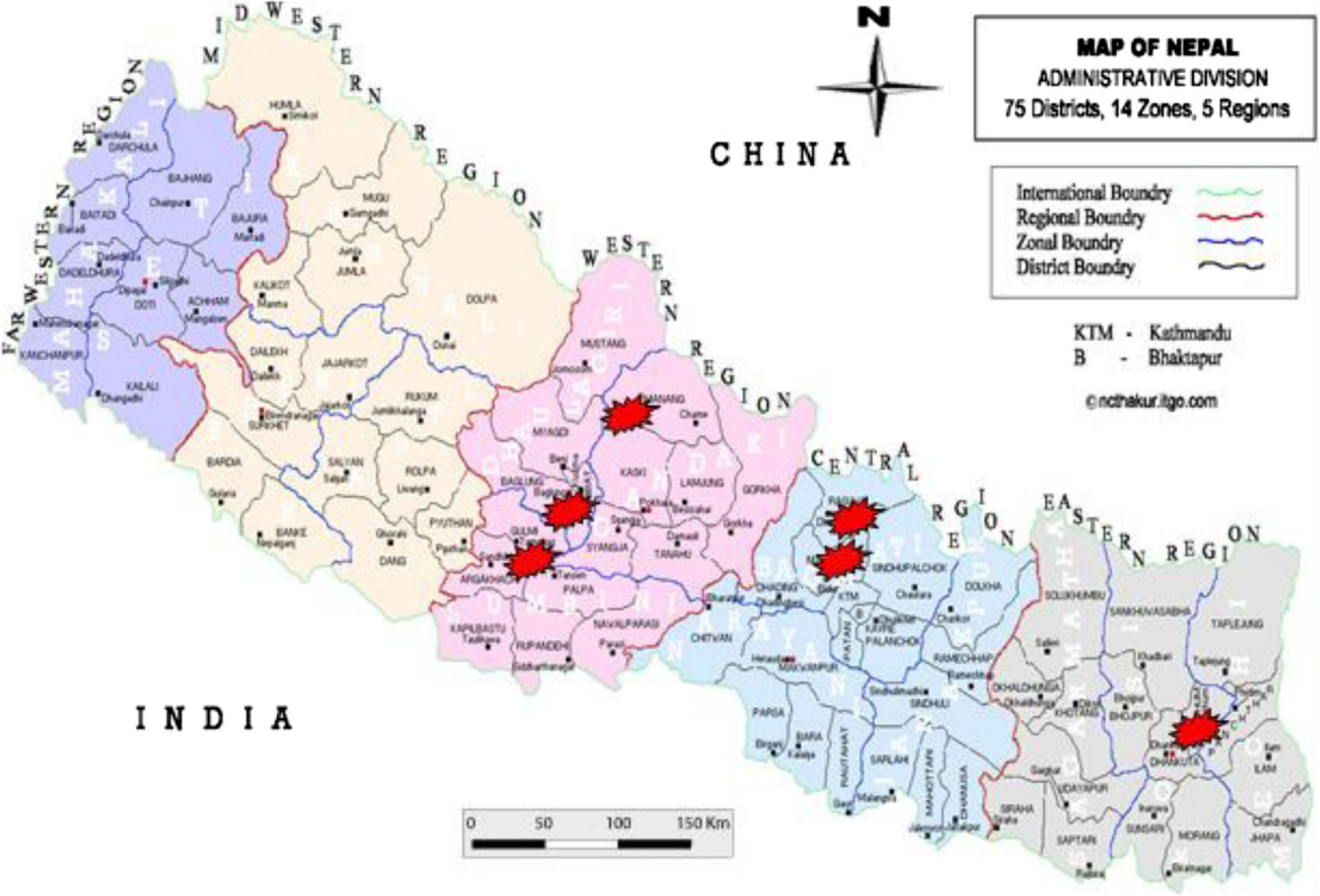
Map of Nepal showing location of participating laboratories in various development regions

Nepal is divided into five development regions. The study was carried out in 12 participating laboratories that are under Antimicrobial Resistance Surveillance network. The study was conducted in three development regions: central, eastern, and western development regions and covered six districts. In the central development region, there were five surveillance sites in Kathmandu district (National Public Health Laboratory (NPHL), Bir Hospital, Sukraraj Tropical Hospital, Kanti Children Hospital, Teaching Hospital) and two in Lalitpur district (Patan Hospital and KIST hospital). In the western development region, Western Regional Hospital and Manipal Teaching Hospital fall in Kaski district whereas Lumbini Zonal Hospital falls in Rupandehi district and United Mission Hospital falls in Palpa district. Only one site BPKIHS falls in Morang district of the eastern development region (Fig. 1).

### Selection of surveillance sites

Surveillance sites were selected preferring large private/government hospital laboratories from various development regions where the diagnostic facilities were adequate and the probability of patient inflow during outbreaks was higher.

### Sample population and data collection

Diarrheal samples were collected from individual patients referring to the private or governmental laboratories. A questionnaire for each subject was filled at respective laboratories mentioning basic details of the patient, age (months), sex, location of residence, and travel. A history from all patients was also recorded, including duration of illness prior to admission to hospital (days), fever (defined as a prolonged temperature >37.5 °C), abdominal discomfort, vomiting, watery diarrhea (defined as three or more loose bowel movements during a 24-h period), bloody or mucoid diarrhea (defined as >3 loose stools with obvious blood or mucus), estimated number of episodes of diarrhea before attending hospital, and if there was any known pretreatment with antimicrobials.

### Sources of *Shigellae*

Isolates recovered from all 12 sites of Antimicrobial Resistance surveillance network in Nepal during 2003–2015 were reported to NPHL and were included in the study.

### Laboratory methods

Stool specimens received at respective laboratories were cultured for enteric pathogens, including *Shigella* spp. by standard methods [17]. Sample was streaked onto MacConkey agar and a selective medium (either deoxycholate citrate agar or xylose lysine deoxycholate agar or *Salmonella*-*Shigella* agar) (all media by HiMedia, India) by the quadrant isolation technique [18]. The plates were incubated at 37 °C for 24 h. Isolates were identified by Gram’s staining, colony characteristics, and biochemical tests.

After identification of isolates, they were subcultured onto nutrient agar slants in screw capped tube, incubated overnight and then transported to NPHL in a cold box. All reported isolates of *Shigella* were re-confirmed by serotyping using commercially available antisera from Denka Seiken, Japan, and were preserved in tryptic soy broth with 20 % glycerol at −75 °C for further use. Antibiotic susceptibility test was performed for all the identified *Shigella* isolates by Standard Kirby Bauer’s disc diffusion technique. The antibiotics used for analysis were ampicillin (Amp-10 mcg), cotrimoxazole (Sxt-25 mcg), nalidixic acid (NA-30 mcg), ciprofloxacin (Cip-5 mcg), mecillinam (Mec), and ceftriaxone (CRO-5 mcg).

### Statistical methods

Data obtained were analyzed using SPSS software for windows version 18. Data on variables like shigella serotypes, age group, and sex were calculated as percentages and compared using chi-square test. *P* < 0.05 was considered to be statistically significant. Duplicate samples were counted as one during data entry.

## Results

**Fig. 2:**
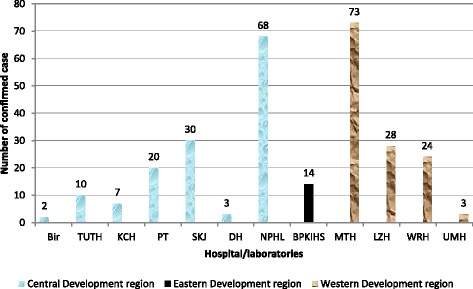
Region wise reported cases of total *Shigella* isolates (*n* = 232)

A total of 332 *Shigella* isolates were reported in a period of 2003–2015 from 12 participating laboratories of Antimicrobial Resistance surveillance network in Nepal. The highest number was reported in 2005. Peak isolation was seen in post monsoon season, i.e., September (Fig. 2).

*Shigella dysenteriae* was the prevalent species in Nepal in the years 2003–2004 but since 2005, *S. flexneri* replaced it and became the most prevalent species. A marked increase in the number of *S. sonnei* (*N* = 6) was noticed in the year 2005 which is highest reported till date. In contrast, no isolate of *S. boydii* was reported since 2009 till 2014 where one isolate was reported. Since 2008, few cases of unidentified *Shigella* isolates were also reported (Table 1).

**Table 1:** Annual distribution of different serotypes of *Shigella* (2003–2015)

Year	*S. dysentriae N* (%)	*S. flexneri N* (%)	*S. sonnei N* (%)	*S. boydii N* (%)	Unidentified *Shigella* spp.	Total
2003	13 (52 %)	7 (28 %)	2 (8 %)	3 (12 %)	–	25
2004	28 (63.7 %)	12 (27.3 %)	2 (4.5 %)	2 (4.5 %)	–	44
2005	10 (19.6 %)	31 (60.8 %)	6 (11.8 %)	4 (7.8 %)	–	51
2006	6 (26.1 %)	14 (60.9 %)	2 (8.7 %)	1 (4.3 %)	–	23
2007	10 (27 %)	22 (59.5 %)	3 (8.1 %)	2 (5.4 %)	–	37
2008	2 (11.8 %)	8 (47.2 %)	1 (5.8 %)	1 (5.8 %)	5 (29.4 %)	17
2009	8 (40 %)	5 (25 %)	1 (5 %)	1 (5 %)	5 (25 %)	20
2010	5 (50 %)	4 (40 %)	–	–	1 (10 %)	10
2011	2 (18.2 %)	5 (45.4 %)	2 (18.2 %)	–	2 (18.2 %)	11
2012	2 (18.2 %)	6 (54.5 %)	3 (27.3 %)	–	–	11
2013	5 (16.1 %)	21 (67.7 %)	3 (9.7 %)	–	2 (6.5 %)	31
2014	1 (4 %)	12 (48 %)	5 (20 %)	1 (4 %)	6 (24 %)	25
2015	3 (11.1 %)	19 (70.4 %)	4 (14.8 %)	–	1 (3.7 %)	27
Total	95 (28.6 %)	166 (50 %)	34 (10.2 %)	15 (4.5 %)	22 (6.6 %)	332

**Fig. 3:**
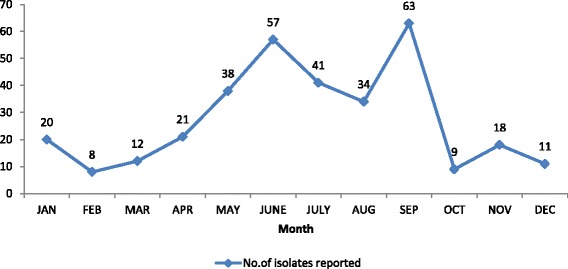
Seasonal distribution of total reported cases (*n* = 232)

Region wise distribution of reported isolates showed that 18 isolates of *Shigella* were reported from one laboratory in the eastern development region, 188 isolates were reported from seven participating laboratories in the central development region, and 126 were reported from four participating laboratories in the western development region (Fig. 3).

**Fig. 4:**
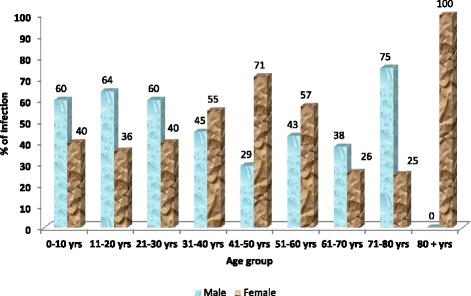
Percentage of laboratory-confirmed *Shigella* infection reported to NPHL, by age group and sex (*n* = 196 with age and sex information reported)

Of the total, 24.5 % of cases were demonstrated in children below 10 years followed by 21–30 years age group (22.9 %) and 11–20 years age group (18.5 %). Significant difference was not noted (*p* = 0.059) in age wise distribution of cases over the years. *Shigella* infection was reported higher among male in age groups below 30 years and 71–80 years but higher cases were reported in females of age groups 31–70 years and 80+ years (Fig. 4).

**Fig. 5:**
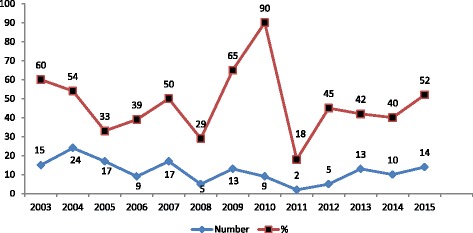
Year wise distribution of MDR isolates (in number and percentage)

The antimicrobial susceptibility profile of *Shigella* isolates in the years 2003–2015 is shown in Table 2. Different serotypes of *Shigella* exhibited various resistance patterns. *S. dysentriae* showed varying resistance to ampicillin (80–100 %), co-trimoxazole (20–100 %), and nalidixic acid (20–100 %). Likewise, *S. flexneri* also exhibited varying resistance to ampicillin (80–100 %), co-trimoxazole (58–100 %), and nalidixic acid (0–100 %). Until 2003, only *S. dysentriae* strains exhibited resistance to ciprofloxacin; however, 50 % of the *S. flexneri* isolates were resistant to ciprofloxacin by 2004. By 2012, *S. dysentriae* strains were found susceptible to ciprofloxacin in contrast to *S. flexneri*, which exhibited highest resistance. Among *S. sonnei* and *S. boydii* serotypes, resistance to ampicillin, cotrimoxazole, and nalidixic acid kept varying. Only three *S. sonnei* isolates were found resistant to ciprofloxacin while no ciprofloxacin-resistant *S. boydii* was reported. All the serotypes of *Shigella* were 100 % resistant to mecillinam since 2007; so by 2010, mecillinam was no longer included in antimicrobial testing of *Shigella.* Among the serotypes, resistance to ceftriaxone was first reported in *S. flexneri* (10–33 %) followed by non-typable *Shigella* spp. with around 40 % resistance; 22.2% of the total isolates tested were resistant to all three antimicrobials. Figure 5 demonstrates the cumulative antibiogram of various species of *Shigella*.

**Table 2:** Antimicrobial susceptibility profile of various species of *Shigella* (2003–2015)

	*N* (%) resistance to							
Year	Species	Number tested	Ampicillin	Ciprofloxacin	Nalidixic acid	Cotrimoxazole	Mecillinam	Ceftriaxone
2003	*S. dysentriae*	13	11 (85)	8 (62)	10 (77)	9 (69)	5 (38)	0
	*S. flexneri*	7	7 (100)	0	0	6 (86)	2/6 (33)^a^	0
	*S. boydii*	3	2 (66)	0	1 (33)	2 (66)	2 (66)	0
	*S. sonnei*	2	1 (50)	0	2 (100)	2 (100)	1 (50)	0
2004	*S. dysentriae*	28	25 (89)	13 (46)	14 (50)	18 (64)	20/27 (74)^a^	0
	*S. flexneri*	12	9 (75)	6 (50)	6 (50)	8 (67)	4 (33)	0
	*S. boydii*	2	1 (50)	0	0	1 (50)	0	0
	*S. sonnei*	2	0	0	2 (100)	2 (100)	0	0
2005	*S.dysentriae*	10	8 (80)	6/9 (66)^a^	7 (70)	8 (80)	2/8 (25)^a^	0
	*S. flexneri*	31	18/30 (60)^a^	0	17/28 (59)^a^	21 (68)	13 (42)	0
	*S. boydii*	6	4 (66)	0	3 (50)	0	4 (66)	0
	*S. sonnei*	4	1 (25)	0	2 (50)	2 (50)	0	0
2006	*S. dysentriae*	6	4/5 (80)^a^	0	4/5 (80)^a^	2/5 (40)^a^	4 (66)	0
	*S. flexneri*	14	8/13 (62)^a^	3/12 (25)^a^	4/10 (40)^a^	7/12 (58)^a^	14 (100)	0
	*S. boydii*	2	0	0	0	0	0	0
	*S. sonnei*	1	S	R	R	S	S	S
2007	*S. dysentriae*	10	5/6 (83)^a^	4 (40)	2 (20)	2 (20)	10 (100)	0
	*S. flexneri*	22	17/21 (81)^a^	12/21 (57)^a^	21 (95)	19/21 (90)^a^	22 (100)	2/20 (10)^a^
	*S. boydii*	3	3 (100)	0	0	0	3 (100)	0
	*S. sonnei*	2	0	0	0	0	2 (100)	0
2008	*S. dysentriae*	2	0	2 (100)	2 (100)	0	2 (100)	0
	*S. flexneri*	8	8 (100)	3/5 (60)^a^	8 (100)	4/5 (80)^a^	8 (100)	0
	*S. sonnei*	2	2 (100)	0	2 (100)	0	2 (100)	0
	*Shigella* spp.	5	3 (60)	1/4 (25)^a^	2/4 (50)^a^	3/4 (75)^a^	5 (100)	1/3 (33)^a^
2009	*S. dysentriae*	8	8 (100)	2 (25)	7 (88)	7 (88)	8 (100)	0
	*S. flexneri*	6	4/5 (80)^a^	6 (100)	4/5 (80)^a^	6 (100)	6 (100)	2 (33)
	*S. sonnei*	1	R	S	R	S	R	S
	*Shigella* spp.	5	3 (60)	3 (60)	4 (80)	4 (80)	5 (100)	2 (40)
2010	*S. dysentriae*	5	5 (100)	1 (20)	5 (100)	5 (100)	–	0
	*S. flexneri*	4	4 (100)	1 (25)	4 (100)	4 (100)	–	0
	*Shigella* spp.	1	R	S	R	R	–	S
2011	*S. dysentriae*	2	2 (100)	1 (50)	1 (50)	2 (100)	–	0
	*S. flexneri*	5	3 (60)	0	0	3/4 (75)^a^	–	0
	*S. sonnei*	2	1 (50)	0	2 (100)	0	–	0
	*Shigella* spp.	2	2 (100)	0	2 (100)	2 (100)	–	0
2012	*S. dysentriae*	4	4 (100)	0	3 (75)	3 (75)	–	0
	*S. flexneri*	7	7 (100)	4/6 (66)^a^	3/4 (75)^a^	4/6 (67)^a^	–	2/6 (33)^a^
	*S. sonnei*	3	0	0	1 (33)	3 (100)	–	0
2013	*S. dysentriae*	5	5 (100)	0	4 (80)	3 (60)	–	0
	*S. flexneri*	19	15/16 (93)^a^	12/17 (71)^a^	16/18 (89)^a^	14 (74)	–	5/18 (27)^a^
	*S. sonnei*	3	3 (100)	2 (66)	2 (66)	2 (66)	–	0
	*Shigella* spp.	3	3 (100)	0	3 (100)	3 (100)	–	0
2014	*S. dysentriae*	1	S	R	S	S	–	S
	*S. flexneri*	12	7/11 (64)^a^	8 (66)	11 (92)	7 (58)	–	0
	*S. sonnei*	5	3 (60)	5 (100)	5 (100)	5 (100)	–	0
	*S. boydii*	1	S	R	R	R	–	S
	*Shigella* spp.	6	6 (100)	1/5 (20)^a^	3/4 (75)^a^	3 (50)	–	0
2015	*S. dysentriae*	3	0	0	3 (100)	2 (75)	–	0
	*S. flexneri*	19	16/18 (88)^a^	9 (47)	15 (79)	17 (89)	–	3 (16)
	*S. sonnei*	4	3 (75)	4 (100)	4 (100)	4 (100)	–	0
	*Shigella* spp.	1	R	S	R	R	–	S

^a^The number in which antibiotics were incorporated, if less than the total number of isolates

The prevalence of multidrug-resistant (resistance to two or more classes of antibiotics) *Shigella* isolates is demonstrated in Fig. 5. The prevalence of MDR increased significantly in 2010 as compared to 2003. On the basis of their pattern of antimicrobial resistance to the six antibiotics used in the study, the reported *Shigellae* were grouped into seven phenotypes (Table 3). Among the *Shigella* serotypes, high prevalence of MDR was observed in *S. dysentriae* isolates occupying 52 % of the total isolates.

**Table 3:** Multidrug-resistant phenotypes of *Shigella*

MDR phenotype	Number (%) of isolates exhibiting resistance				
	*S. dysentriae*	*S. flexneri*	*S. boydii*	*S. sonnei*	*Shigella* spp.
Amp/Cip or NA/Mec	1 (1.9 %)	3 (5.2 %)	2 (50 %)	–	–
Amp/Mec/Ts	5 (9.4 %)	3 (5.2 %)	–	–	–
Mec/Cip or NA/Ts	2 (3.8 %)	3 (5.2 %)	–	1 (10 %)	–
Amp/NA or Cip/Ts	25 (47.2 %)	36 (62.1 %)	1 (25 %)	9 (90 %)	3 (50 %)
Amp/NA or Cip/Ts/Mec	20 (37.7 %)	5 (8.6 %)	–	–	3 (50 %)
Amp/NA or Cip/Ts/Mec/CRO	–	1 (1.7 %)	1 (25 %)	–	–
Amp/NA or Cip/Ts/CRO	–	7 (11.9 %)	–	–	–
Total number MDR (%)	53 (55.8 %)	58 (34.9 %)	4 (26.6 %)	10 (29.4 %)	6 (27.3 %)

## Discussion

Shigellosis still accounts for a significant proportion of mortality and morbidity, especially in developing countries [19]. The present study, covering 13 years surveillance (2003–2015), demonstrates the changing serotype and trend of antimicrobial resistance of *Shigella* isolates in Nepal.

An analysis of serotypes revealed that *S. dysenteriae* was the prevalent species in Nepal in the years 1999–2004 but a change in serotype in *Shigella* species was noticed during 2005. The changing patterns in the distribution of *Shigella* serogroups and serotypes have been reported from time to time [20–23]. In our study, *S. flexneri* remained the most prevalent serotype in Nepal since 2005 which is in accordance with many recent studies in Nepal and other developing countries [9, 15, 17, 24–27] but dissimilar to some other studies [28–30]. It has been hypothesized that these changes may be due to varying behavourial and environmental factors such as reduction of overall malnutrition, use of safe drinking water instead from tubewells or pipes, and improved sanitary facilities for defecation [31].

In our study, like in other studies, the seasonal tendency of shigellosis was summer-monsoon [9, 15]. This may be because during this period, there is a climatic variation due to which the sources of drinking water gets rapidly contaminated. Males were more commonly infected as compared to females as in other studies made by Khan et al. and Taneja [15, 32]. This male preponderance may be due to greater chances of exposure to male population who go out of the house much frequently than females for eating food from restaurants and street vendors. The susceptible age group in our study, like in other studies, was below 10 years [12, 15, 25]. In our study, very few cases were reported from children below 5 years. This may be because the laboratories in the surveillance network are limited; many cases may be referred to private institutions which are not included in the surveillance network. Besides that, other factors like incomplete information on age (16 % missing) available from participating laboratories, inclusion of only one children’s hospital in the surveillance and low *Shigella* isolation rate may have contributed to under reporting of true number of victims.

Treatment of shigellosis depends on appropriate antimicrobial therapy. A variety of antibiotics are effective for treatment of shigellosis, although options are becoming limited due to globally emerging drug resistance. The present study also shows high resistance among all *Shigella* species to the first line low cost drugs commonly used in the treatment of shigellosis like Ampicillin, Cotrimoxazole and Nalidixic acid. Considering resistance pattern in different groups, *S. flexneri* and *S. dysentriae*, the two most common serotypes revealed similar pattern in resistance with slightly higher range in *S. flexneri*. However, the difference was not statistically significant. Similar studies conducted by Khan et al. reveals that *S. flexneri* exhibited increased resistance to ampicillin and nalidixic acid as compared to *S. dysentriae* whereas high resistance was observed against ciprofloxacin and cotrimoxazole in *S. flexneri* as compared to *S. dysentriae* [15]*.* This finding however does not agree with some previous studies done by Wilson et al. and Kansakar et al. [9, 12] in Nepal which demonstrates high resistance in *S. dysentriae*. Among remaining serotypes, untypable *Shigella* spp. exhibited high resistance to all antimicrobials except ciprofloxacin as compared to *S. sonnei* and *S. boydii. S. boydii* exhibited least resistance to all antimicrobials tested. This finding is also in accordance to studies made by Khan et al. [15] but contradictory to findings by Kansakar et al. [9] which reveals *S. sonnei* as the most susceptible serotype. Emerging resistance to ceftriaxone in *S. dysentriae*, *S. flexneri*, and *Shigella* spp. is noted which raises concern for the use of third-generation cephalosporins as last therapy of treatment.

This difference in antimicrobial resistance among various serotypes may be attributed to change in time period of the studies. Studies conducted during 2004/2005 showed contradictory result in terms of prevalent serotype and resistance profile among serotypes as compared to that of 2013/2014 which seems obvious.

MDR (resistance to three or more than three antibiotics) was found in 46.1 % of total isolates, and the most common MDR profile was ampicillin, nalidixic acid, and cotrimoxazole. This finding is very low as compared to 90.2 % reported by Reema et al. However, the MDR profile is similar [33].

### Limitations

Data represented from our 12 surveillance sites may not be representative of the sick population at large in the community. Lack of total number of reported cases and inadequate data management systems also limited the results of our study.

## Conclusions

With increasing resistance to first-line agents along with ciprofloxacin, only ceftriaxone remains the drug of choice; however, increasing MDR along with resistance to third-generation cephalosporins as last resort creates an alarming situation warranting proper usage of antimicrobial agents and its continuous monitoring.

## Abbreviations

Amp: Ampicillin
BPKIHS: B. P. Koirala Institute of Health Sciences
Cip: Ciprofloxacin
CRO: Ceftriaxone
KIST: KIST Medical College and Teaching Hospital
MDR: Multidrug resistant
Mec: Mecillinam
NA: Nalidixic acid
NPHL: National Public Health Laboratory
Ts: Cotrimoxazole
WHO: World Health Organization
